# ERK1/2-CEBPB Axis-Regulated hBD1 Enhances Anti-Tuberculosis Capacity in Alveolar Type II Epithelial Cells

**DOI:** 10.3390/ijms25042408

**Published:** 2024-02-18

**Authors:** Yaoxin Chen, Zhenyu Han, Sian Zhang, Honglin Liu, Ke Wang, Jieyu Liu, Feichang Liu, Shiyun Yu, Na Sai, Haiyan Mai, Xinying Zhou, Chaoying Zhou, Qian Wen, Li Ma

**Affiliations:** 1 Institute of Molecular Immunology, School of Laboratory Medicine and Biotechnology, Southern Medical University, Guangzhou 510515, China; 15622607177@163.com (Y.C.); han19960809@i.smu.edu.cn (Z.H.); 15552272281@163.com (S.Z.); 18638875241@163.com (H.L.); wangke949@163.com (K.W.); ljy2020trna@163.com (J.L.); solededlfc@163.com (F.L.); 13049117035@163.com (S.Y.); m13150826890@163.com (N.S.); 13416369212@163.com (H.M.); zxyforever@smu.edu.cn (X.Z.); zhouchaoying100@163.com (C.Z.); 2 Key Laboratory of Infectious Diseases Research in South China (Southern Medical University), Ministry of Education, Guangzhou 510515, China

**Keywords:** *Mycobacterium tuberculosis*, alveolar type II epithelial cells, human β-defensin 1, CEBPB

## Abstract

Tuberculosis, caused by *Mycobacterium tuberculosis* (Mtb), remains a global health crisis with substantial morbidity and mortality rates. Type II alveolar epithelial cells (AEC-II) play a critical role in the pulmonary immune response against Mtb infection by secreting effector molecules such as antimicrobial peptides (AMPs). Here, human β-defensin 1 (hBD1), an important AMP produced by AEC-II, has been demonstrated to exert potent anti-tuberculosis activity. HBD1 overexpression effectively inhibited Mtb proliferation in AEC-II, while mice lacking hBD1 exhibited susceptibility to Mtb and increased lung tissue inflammation. Mechanistically, in A549 cells infected with Mtb, STAT1 negatively regulated hBD1 transcription, while CEBPB was the primary transcription factor upregulating hBD1 expression. Furthermore, we revealed that the ERK1/2 signaling pathway activated by Mtb infection led to CEBPB phosphorylation and nuclear translocation, which subsequently promoted hBD1 expression. Our findings suggest that the ERK1/2-CEBPB-hBD1 regulatory axis can be a potential therapeutic target for anti-tuberculosis therapy aimed at enhancing the immune response of AEC-II cells.

## 1. Introduction

Tuberculosis, caused by *Mycobacterium tuberculosis* (Mtb) infection, is the deadliest infectious disease worldwide. Furthermore, due to the previously ongoing COVID-19 pandemic, tuberculosis prevention and control services have been severely affected. Additionally, drug-resistant tuberculosis has become increasingly serious, with 3.6% of new cases and 18% of previously treated cases being multidrug-resistant or rifampicin-resistant tuberculosis (MDR/RR-TB), of which 20% are extensively drug-resistant tuberculosis (XDR-TB) [1]. Therefore, in-depth research on the immune mechanisms of Mtb infection and the development of novel immunotherapies for tuberculosis have become urgent priorities.

Tuberculosis is a respiratory-transmitted disease. The abundant hydrophobic lipids on the surface of Mtb allow it to attach to tiny droplets and rapidly reach the lungs, enabling it to bypass the killing mechanisms of the natural immune system and establish infection in the lungs [2]. The human lungs primarily consist of pulmonary epithelial cells, which are the first cellular population encountered by invading Mtb. Pulmonary alveolar epithelial cells not only form a dense barrier for exogenous pathogens but also promote the phagocytosis of Mtb by alveolar macrophages through the expression of surface receptors and the secretion of surfactant proteins SP-A and SP-D [3]. This helps maintain lung homeostasis and relative sterility. Among the single-layered flat epithelium that constitutes the alveolar wall, type II alveolar epithelial cells (AEC-II) account for 14–16% of the total alveolar epithelial cells [4]. AEC-II cells possess stemness and can proliferate to form new AEC-II cells, as well as differentiate into type I alveolar epithelial cells (AEC-I) to repair the damaged alveolar epithelial barrier caused by Mtb infection [5]. AEC-II cells perform various anti-infective immune functions in the alveoli. In addition to expressing various pattern recognition receptors [6], they can also phagocytose Mtb in the early stages of infection [7], process mycobacterial antigens via the MHC-II pathway, and present antigens to T cells, thereby stimulating the memory immune response [8]. Additionally, AEC-II cells play a significant role in the release of cytokines for cell cross-talk [9] and directly exhibit antimicrobial activity through the expression of antimicrobial peptides (AMPs) and other effector molecules [7,10,11].

AMPs are small-molecule peptides composed of 10–50 amino acids. They are present in various tissues and cells, and exhibit broad-spectrum antimicrobial activity through multiple mechanisms, such as altering cell wall and membrane permeability, promoting intra- and extra-cellular ion exchange, and directly and rapidly killing microorganisms [12]. Due to their ability to target multiple sites, such as creating pores directly on the cell membrane or binding to bacterial cell membrane proteins, peptidoglycan precursor lipid II, AMPs inhibit bacterial cell membrane and cell wall synthesis. Furthermore, due to the cationic nature of AMP, like 1018-K6, it tends to prefer binding to the prokaryotic membrane rather than interacting with the plasma membrane of human cells [13]. Their unique antimicrobial mechanism allows them to bypass microbial resistance induction mechanisms and exert rapid killing effects, minimizing the emergence of drug-resistant strains and even directly killing multidrug-resistant bacteria [14]. Patients with drug-resistant tuberculosis require personalized treatment plans lasting at least 18 months [15], consuming a substantial portion of healthcare budgets and related resources in many endemic countries. Thus, drug-resistant tuberculosis significantly exacerbates the global burden of antimicrobial resistance. Several studies have demonstrated that AMPs such as PR-39 and AZPs can effectively kill MDR or XDR Mtb, with growth inhibition rates exceeding 50% [16]. Rekha et al. found that LL-37 can induce autophagy of macrophages through activating P2RX7 receptor, which in turn enhance the release of cytoplasmic free Ca^2+^, and the subsequent activation of AMPK and PtdIns3K pathways, thereby promoting the intracellular killing of Mtb in human macrophages [17]. Based on the cyclic peptide griselimycin from Streptomyces, Kling A et al. designed and synthesized a novel AMP, cyclohexyl griselimycin, which exhibited potent inhibitory activity against Mtb both in vitro and in vivo by targeting the DNA polymerase sliding clamp DnaN [18]. This suggests that combining AMPs with other conventional treatment methods could be a feasible therapeutic approach.

Defensins are an evolutionarily related family of vertebrate AMPs, characterized by a conserved framework of β-sheet-rich and six disulfide-connected cysteines. In addition to their expression in leukocytes, defensins are also highly expressed in various types of epithelial cells, exerting broad-spectrum antimicrobial activities [19]. hBD1, as a member of the β-defensin family of AMPs, is one of the most important AMPs secreted by epithelial tissues. It is abundant in body fluids such as urine, saliva, and milk [20,21,22]. In extracellular neutral and acidic environments, hBD1 can synergize with lysozyme to combat Staphylococcus aureus [23]. Goldman MJ et al. found that as a constitutively expressed secreted peptide, the gene expression of hBD1 in the airway surface fluid of the human lung is distributed throughout the respiratory epithelial tissue, playing a crucial role in defending against Pseudomonas aeruginosa infections commonly seen in cystic fibrosis patients [24]. hBD1 also exhibits activity against Mtb infection. Fattorini L et al. demonstrated that hBD1 enhances the action of isoniazid and significantly inhibits the growth of H37Rv in in vitro cultures [25]. The addition of hBD1 to in vitro cultures of actively growing H37Rv resulted in the killing of 98% of the bacteria. When co-cultured with infected macrophages, hBD1 upregulates the expression of IFN-γ in macrophages, leading to a more efficient inhibition of intracellular H37Rv survival [26]. Furthermore, hBD1 displays higher killing activity against dormant H37Rv in vitro and within granulomas compared to rifampicin and isoniazid, providing a potential important means for the complete elimination of dormant and drug-resistant MTB infections [26]. However, research on the anti-tuberculosis effects of hBD1 is still limited, and studies on the killing of intracellular Mtb by hBD1 expressed in infected lung epithelial cells are lacking. Considering hBD1’s role as a constitutively expressed defensive antimicrobial effector molecule in AEC-II cells and its direct killing effect on invading pathogens, it is likely to play a role as the first line of defense in the innate immune response against tuberculosis infection. In fact, in our study, we have indeed found that upregulation of hBD1 expression contributes to the inhibition of intracellular mycobacterial survival and dissemination, making it a promising adjunctive anti-tuberculosis agent.

However, the small molecular weight, cationic nature, and sensitivity to salt and pH of AMPs make them prone to hydrolysis and inactivation, resulting in high production costs and difficulties in preservation [27]. Therefore, it may be more effective to enhance the levels of endogenous AMPs by utilizing the inherent regulatory mechanisms of their expression as an immunotherapeutic approach against tuberculosis. Previous reports have shown that the expression of the hBD1 encoding gene, defensin β 1 (*DEFB1*), is regulated by various pathways. For example, epigallocatechin gallate can promote hBD1 levels through the ERK1/2 and p38 pathways [28], while the MEKK1/2-ERK1/2 signaling pathway can inhibit hBD1 levels through the transcription factor MYC [29]. Additionally, the transcription factor PAX2 can bind to the PAX2 homologous sequence in the *DEFB1* promoter to suppress hBD1 levels [30]. However, there have been no reports on the regulation of hBD1 expression during lung epithelial cell responses to Mtb infection.

In this study, we evaluated the anti-Mtb infection effect of hBD1 in human lung epithelial cells and mice first, and performed bioinformatics analysis to predict the transcription factors that may be involved in regulating *DEFB1* transcription. We identified two transcription factors with opposite effects, signal transducer and activator of transcription 1 (STAT1) and CCAAT/enhancer-binding protein beta (CEBPB), and explored the signaling pathways involved in hBD1 expression regulation. Our findings will provide a basis for the development of novel therapeutic strategies that modulate the expression levels of endogenous hBD1 and enhance the efficacy of anti-tuberculosis chemotherapy.

## 2. Results

### 2.1. HBD1 Effectively Inhibits the Proliferation of Mtb In Vitro in AEC-II Cells and In Vivo

**Figure 1:**
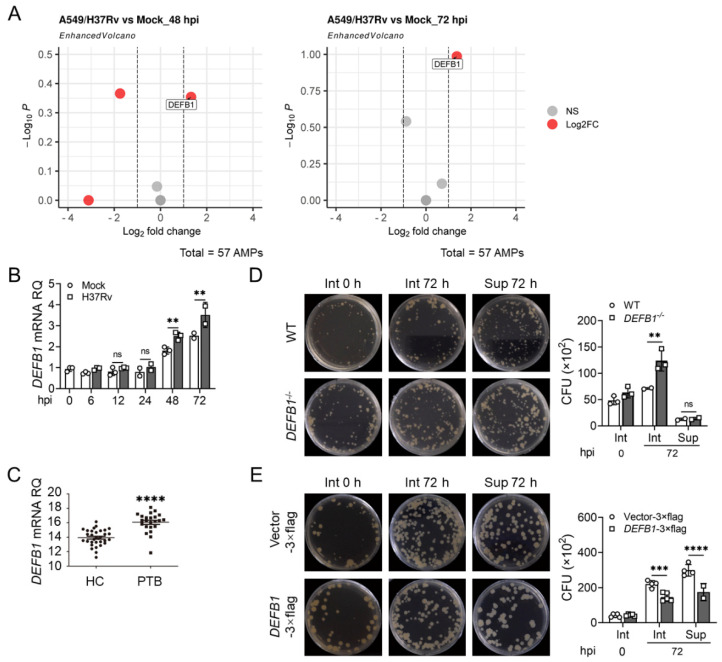
Upregulation of hBD1 in type II alveolar epithelial cells (AEC-II) efficiently suppresses the intracellular proliferation of *Mycobacterium tuberculosis* (Mtb). (**A**) Volcano plots indicating the differential expression of 57 commonly known antimicrobial peptides (AMPs) in A549 cells with or without H37Rv infection at multiplicity of infection (MOI) = 10 for 48 h (**left**) and 72 h (**right**), identified with high throughput RNA sequencing. (**B**) qPCR analysis of *DEFB1* expression in A549 cells infected with H37Rv at MOI = 10 for 72 h. (**C**) qPCR analysis of *DEFB1* expression in peripheral blood mononuclear cells (PBMCs) from healthy controls (HCs) and pulmonary tuberculosis patients (PTB). (**D,E**) colony-forming unit (CFU) assay of the intracellular (Int) and extracellular culture supernatant(Sup) bacterial load in *DEFB1*^−/−^ A549 cells (**D**) or in *DEFB1*-overexpressing A549 cells (**E**) after being infected with H37Rv at MOI = 10 for 72 h. Data are presented as mean ± SD and are representative of at least three experiments with similar observations. ANOVA was used for comparison involving three or more variables. ** *p* < 0.01; *** *p* < 0.001; **** *p* < 0.0001, ns; non-significant.

To explore the role of AMPs in AEC-II cells, we performed transcriptomic analysis of H37Rv-infected A549 cells. Due to the relatively weaker and slower response of epithelial cells to H37Rv infection compared to macrophages and dendritic cells, accompanied with the consideration that the long-lasting expression of AMPs would perform better against the chronic infection of Mtb, cells were collected at 48 h and 72 h but not 24 h post infection. In total, 57 AMPs were identified. Among them, only the gene encoding hBD1, *DEFB1*, elevated most significantly after infection at both 48 h and 72 h post infection (Figure 1A). Additionally, we analyzed the previously reported RNA dataset of whole lung tissues from tuberculosis patients and uninfected individuals (GSE114911) [31], in which *DEFB1* was also found to be upregulated after infection (Figure S1A). Subsequently, we examined the expression of *DEFB1* in H37Rv-infected A549 cells at different time points and observed an upregulation of *DEFB1* expression in a time-dependent manner (Figure 1B, *p* < 0.01). A similar phenomenon was observed in normal human bronchial epithelial cells (BEAS-2B) (Figure S1B, *p* < 0.001), as well as in peripheral blood mononuclear cells (PBMCs) from tuberculosis patients (Figure 1C, *p* < 0.0001). These results suggest hBD1 may be associated with the cellular response of epithelial cells against Mtb infection.

**Figure 2:**
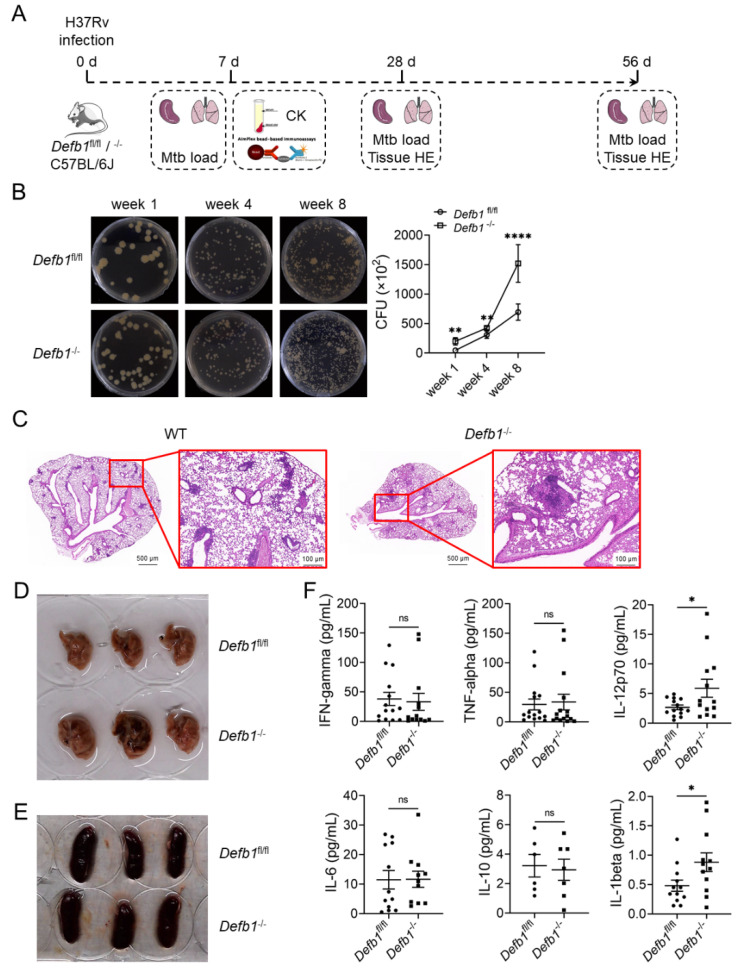
*Defb1*^−/−^ mice exhibits an increased Mtb load of and exacerbates pulmonary inflammation. (**A**,**B**) CFU assay of the bacterial load in the lung of *Defb1^−/−^* mice at 1-, 4- and 8- weeks post H37Rv infection. (**C**,**D**) Histological examination with Hematoxylin and eosin (H&E) staining (**C**) and photographic observation (**D**) of the lung in WT (*Defb^fl/fl^*) and *Defb1^−/−^* mice at 8 weeks post H37Rv infection. (**E**) Photographic observation of the spleen in WT (*Defb^fl/fl^*) and *Defb1^−/−^* mice at 4 weeks post H37Rv infection. (**F**) Luminex assays of serum cytokines in WT and *Defb1^−/−^* mice at 1 week post H37Rv infection. Data are presented as mean ± SD and are representative of at least three experiments with similar observations. ANOVA was used for comparison involving three or more variables. * *p* < 0.05; ** *p* < 0.01; **** *p* < 0.0001, ns; non-significant.

To investigate the antimicrobial effect of hBD1 on Mtb infection, hBD1 was knocking down using CRISPR-Cas9, which resulted in an increase in intracellular bacterial load in A549 cells (Figure 1D, *p* < 0.01; Figure S1C,D, *p* < 0.05). Moreover, overexpressing hBD1 in AEC-II cell lines led to a decrease in intracellular and extracellular bacterial load in both A549 and BEAS-2B cells infected with H37Rv (Figure 1E, *p* < 0.0001; Figure S1E–G, *p* < 0.0001). To further demonstrate the anti-tuberculosis effect of hBD1 in vivo, we constructed *Defb1*^−/−^ mice in which the gene coding the murine counterpart of hBD1 [32,33] was knocked out (Figure 2A and Figure S1H). Upon H37Rv infection, *Defb1^−/−^* mice exhibited a significant increase in lung bacterial load (Figure 2B, *p* < 0.0001). Histological analysis of the tissues displayed evident destruction and an increased infiltration of inflammatory cells, indicating the presence of chronic inflammation. (Figure 2C,D). However, the impact of *Defb1* knockout on the spleen was marginal (Figure 2E), and the cytokine levels also showed no significant change (Figure 2F, *p* < 0.05) except the slight upregulation of IL-1β and IL-17p70, consistent with the certain immunoregulatory activities of hBD1 previously reported [34]. The results above indicating that hBD1 primarily exerts its anti-tuberculosis immune effect in the lung where pulmonary epithelial cells are the main cellular component.

### 2.2. Transcription Factor Prediction for the Regulation of hBD1 Expression

**Figure 3:**
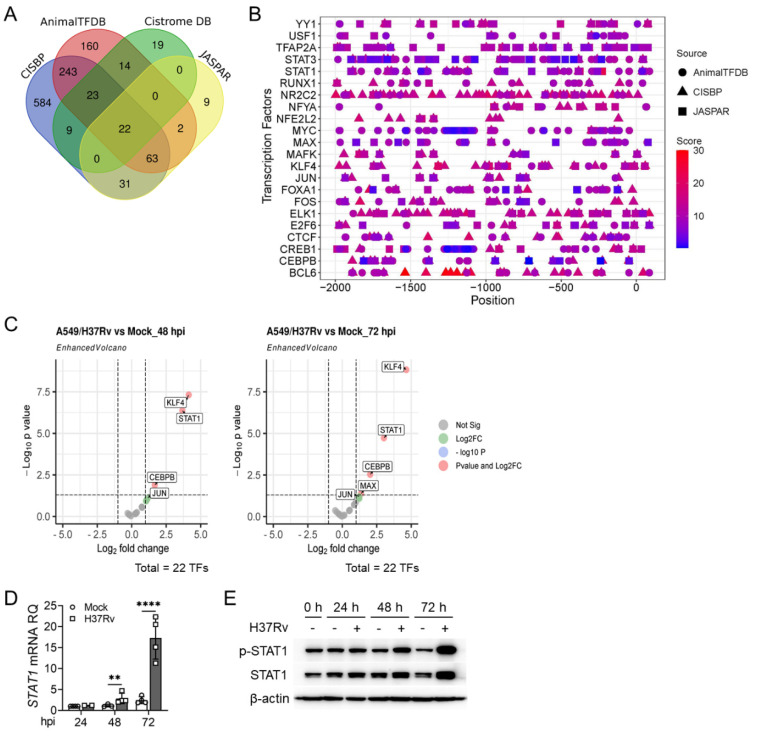
Prediction of transcription factors regulating hBD1 expression. (**A**,**B**) Venn analysis of transcription factors regulating hBD1 expression predicted with databases including CISBP, AnimalTFDB, Cisreome DB, and JASPAR (**A**) Their predicted binding sites on the *DEFB1* gene promoter were displayed as heatmaps using the ggplot2 package in R (Version 4.2.3), following extraction and normalization of the prediction scores (**B**). (**C**) Volcano plots indicating the differential expression of the predicted 22 transcription factors in A549 cells with or without H37Rv infection at MOI = 10 for 48 h (**left**) and 72 h (**right**) identified with high-throughput RNA sequencing. (**D**,**E**) qPCR (**D**) and Western blot (**E**) analysis of STAT1 expression in A549 cells infected with H37Rv at MOI = 5 for 72 h. Data are presented as mean ± SD and are representative of at least three experiments with similar observations. ANOVA was used for comparison involving three or more variables. ** *p* < 0.01; **** *p* < 0.0001.

Due to the susceptibility of AMPs to hydrolysis and inactivation during storage, the cost of synthesizing AMPs in vitro is high. Therefore, we aimed to explore the transcriptional regulatory mechanisms of hBD1 expression in AEC-IIs for developing novel strategies to enhance host anti-tuberculosis immune response by increasing the expression of endogenous hBD1. We combined the use of transcription factor databases including JASPAR, AnimalTFDB, Cisreome DB, and CISBP, to predict the transcription factors of *DEFB1*. Venn analysis showed 22 transcription factors predicted by all four databases (Figure 3A). Furthermore, through extracting and normalizing the prediction scores, and using the ggplot2 package in R (Version 4.2.3) to generate heatmaps [35], binding sites for the these transcription factors on the *DEFB1* promoter were also predicted (Figure 3B), suggesting their potential involvement in the transcriptional regulation of *DEFB1*. Subsequently, we analyzed the expression levels of these 22 transcription factors in A549 cells before and after H37Rv infection using our transcriptome sequencing data, and found that the transcription factors *STAT1*, *CEBPB*, *KFL4*, *JUN*, and *MAX* were upregulated after H37Rv infection (Figure 3C), similar to *DEFB1* expression, suggesting their potential involvement in the transcriptional regulation of *DEFB1*. Among them, STAT1 is one of the important downstream transcription factors of IFN-γ, and it is well known that the IFN-γ/STAT1 pathway plays a crucial protective role in the immune response against Mtb infection [36,37,38]. Therefore, STAT1 may be the transcription factor that promotes *DEFB1* gene transcription in Mtb-infected lung epithelial cells.

### 2.3. STAT1 Downregulates DEFB1 Expression in AEC-II Cells

**Figure 4:**
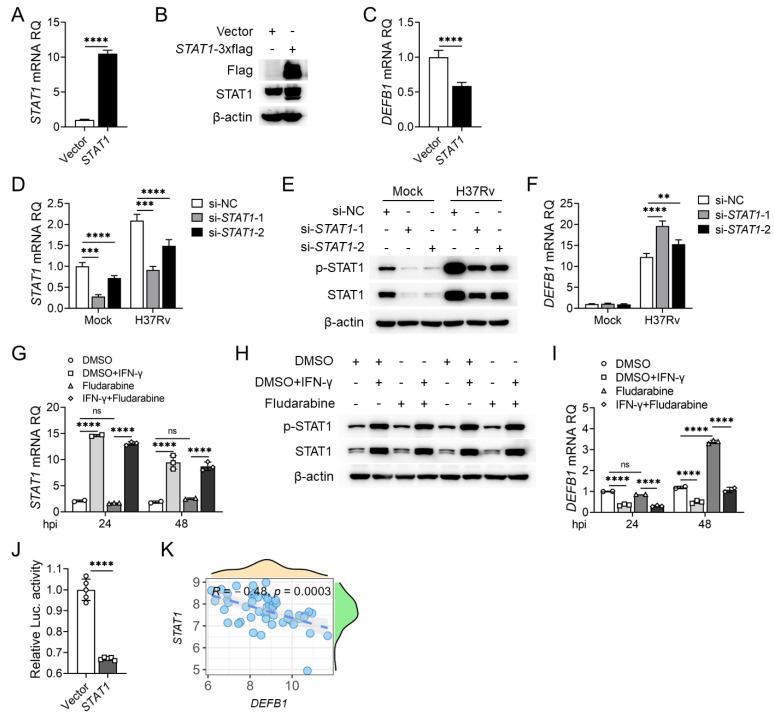
STAT1 downregulates *DEFB1* expression in AEC-II cells. (**A**–**C**) qPCR and Western blot analysis of STAT1 (**A**,**B**) and *DEFB1* (**C**) expression in A549 cells overexpressing STAT1 for 48 h. (**D**–**F**). qPCR and Western blot analysis of STAT1 (**D**,**E**) and *DEFB1* (**F**) expression in *STAT1*-silenced A549 cells after infection with H37Rv at MOI = 10 for 72 h. (**G**–**I**) qPCR and Western blot analysis of STAT1 (**G**,**H**) and *DEFB1* (**I**) expression in A549 cells treated with IFN-γ, Fludarabine or both. (**J**) Luciferase reporter assays of transcriptional regulation of the *DEFB1* promoter by STAT1. (**K**) Analysis of relativity of *DEFB1* and *STAT1* expression in the GSE114911 [31] dataset. Data are presented as mean ± SD and are representative of at least three experiments with similar observations. Simple *t*-tests and ANOVA were used for comparisons involving two and three or more variables, respectively. ** *p* < 0.01; *** *p* < 0.001; **** *p* < 0.0001, ns; non-significant.

To investigate whether the STAT1 pathway is involved in the regulation of *DEFB1* expression in lung epithelial cells, we first examined the expression of STAT1 in H37Rv-infected A549 cells. We found that both RNA and protein levels of STAT1 were upregulated after infection (Figure 3D,E, *p* < 0.0001). However, unexpectedly, overexpression of STAT1 in A549 cells resulted in a downregulation of *DEFB1* expression (Figure 4A–C, *p* < 0.0001), while silencing STAT1 increased *DEFB1* mRNA levels (Figure 4D–F, *p* < 0.0001). Consistently, stimulation of the STAT1 pathway with IFN-γ significantly decreased *DEFB1* mRNA levels, while the STAT1 inhibitor, Fludarabine, promoted *DEFB1* expression (Figure 4G–I, *p* < 0.0001). Luciferase reporter assays confirmed that STAT1 directly bound to the *DEFB1* gene promoter but exerted a transcriptional repressive effect (Figure 4J, *p* < 0.0001). We analyzed the correlation between *DEFB1* and *STAT1* expression in the GSE114911 [31] dataset and found a negative correlation between *STAT1* and *DEFB1* expression in lung tissues from individuals infected with Mtb (Figure 4K). These data indicated that STAT1 acts as a transcriptional repressor of *DEFB1* and is not the transcription factor responsible for the upregulation of *DEFB1* expression in AEC-II cells following Mtb infection. So, which transcription factor plays a positive regulatory role in this process?

### 2.4. CEBPB Promotes DEFB1 Expression in AEC-II Cells

**Figure 5:**
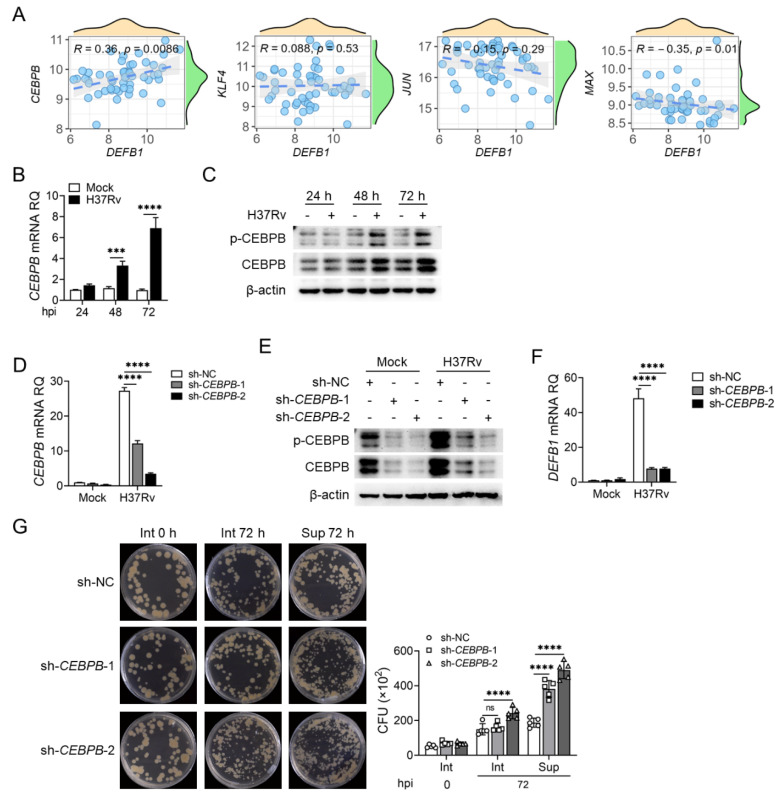
CEBPB functions to promote *DEFB1* expression and suppresses Mtb growth. (**A**) Analysis of relativity of *DEFB1* and CEBPB, KLF4, JUN, and MAX expression in the GSE114911 [31] dataset. (**B**,**C**) qPCR (**B**) and Western blot (**C**) analysis of CEBPB expression in A549 cells infected with H37Rv at MOI = 10 for 72 h. (**D**–**F**) A549 cells were silenced for CEBPB using lentivirus infection, and qPCR and Western blot analysis of CEBPB (**D**,**E**) and *DEFB1* (**F**) expression in A549 cells with CEBPB knockdown and H37Rv infection at MOI = 10 for 72 h. (**G**) CFU assay of the bacterial amounts in and out of A549 cells with CEBPB knockdown and H37Rv infection at MOI = 10 for 72 h. Data are presented as mean ± SD and are representative of at least three experiments with similar observations. Simple *t*-tests and ANOVA were used for comparisons involving two and three or more variables, respectively. *** *p* < 0.001; **** *p* < 0.0001, ns; non-significant.

**Figure 6:**
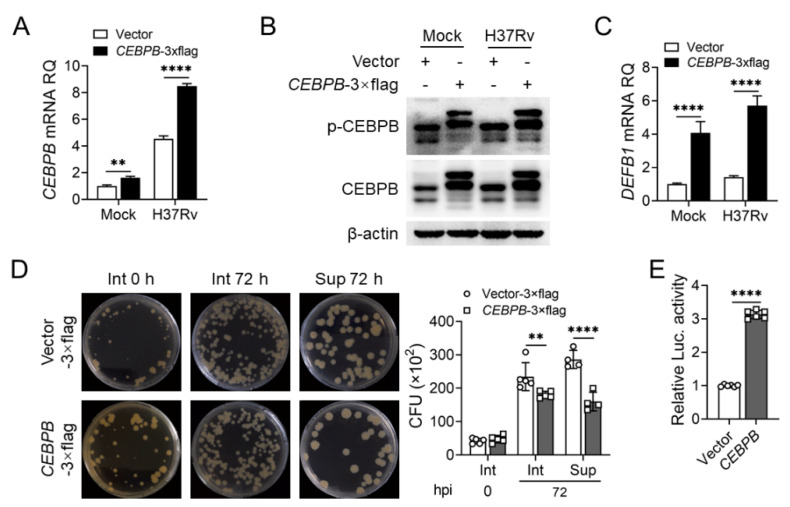
CEBPB promotes *DEFB1* expression to exert anti-tuberculosis effects in AEC-II cells. (**A**–**D**) A549 cells were overexpressed with CEBPB using lentivirus infection, and qPCR and Western blot analysis of CEBPB (**A**,**B**) and *DEFB1* (**C**) expression in A549 cells with CEBPB overexpression and H37Rv infection at MOI = 10 for 72 h. (**D**) CFU assay of the bacterial amounts in and out of A549 cells with CEBPB overexpression and H37Rv infection at MOI = 10 for 72 h. (**E**) Luciferase reporter assays of transcriptional regulation of the *DEFB1* promoter by CEBPB. Data are presented as mean ± SD and are representative of at least three experiments with similar observations. Simple *t*-tests and ANOVA were used for comparisons involving two and three or more variables, respectively. ** *p* < 0.01; **** *p* < 0.0001.

We conducted a similar correlation analysis between the other four transcription factors and *DEFB1* expression in the GSE114911 [31] dataset. Interestingly, only CEBPB expression showed a positive correlation with *DEFB1* (Figure 5A). qPCR and Western blotting validated the results of the data analysis. CEBPB expression was upregulated in AEC-II cells after H37Rv infection, consistent with *DEFB1* expression (Figure 5B,C, *p* < 0.0001; Figure S2A,B, *p* < 0.0001). As expected, silencing CEBPB resulted in decreased *DEFB1* expression and increased both intracellular and extracellular bacterial load in A549 cells (Figure 5D–G, *p* < 0.0001). Conversely, lentiviral overexpression of CEBPB led to an upregulation of *DEFB1* expression and a decrease in intracellular and extracellular H37Rv bacterial load in AEC-II cells (Figure 6A–D, *p* < 0.0001; Figure S2C–F, *p* < 0.0001). Moreover, luciferase reporter assays demonstrated that CEBPB promotes *DEFB1* transcription by directly binding to the *DEFB1* promoter (Figure 6E, *p* < 0.0001). These findings indicate that CEBPB is a positive regulatory transcription factor for *DEFB1*, promoting *DEFB1* expression in AEC-II cells and exerting an anti-tuberculosis effect.

### 2.5. Identification of CEBPB Binding Sites on the DEFB1 Promoter

**Figure 7:**
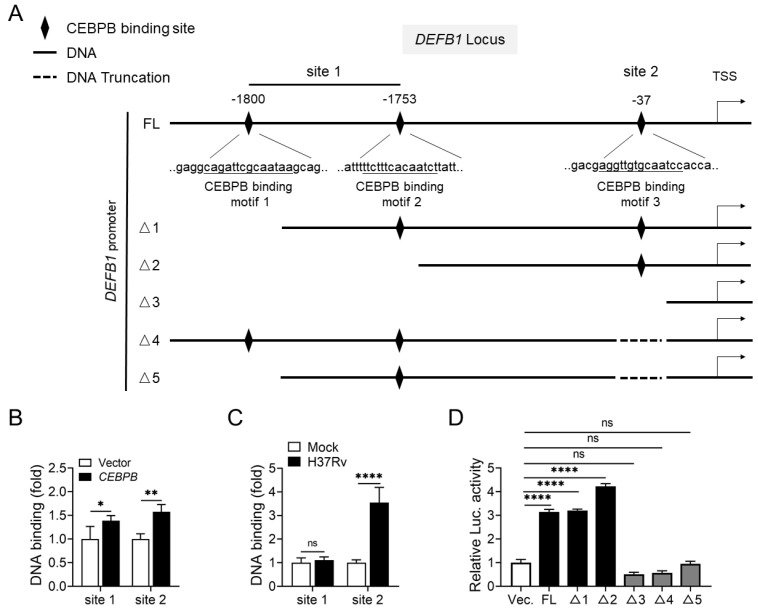
Identification of CEBPB binding sites in the *DEFB1* promoter. (**A**) Schematic diagram of recombinant plasmids carrying full-length or various truncated forms of the *DEFB1* promoter. (**B**) ChIP assays of CEBPB binding to the different regions of the *DEFB1* promoter in A549 cells overexpressing CEBPB. (**C**) ChIP assay of CEBPB binding to the different regions of the *DEFB1* promoter in A549 cells infected with H37Rv at MOI = 10 for 72 h. (**D**) Luciferase reporter assays of transcriptional regulation of full-length or truncated forms of the *DEFB1* promoter by CEBPB in 293T cells transfected with various *DEFB1* promoter expression plasmids. Data are presented as mean ± SD and are representative of at least three experiments with similar observations. ANOVA was used for comparison involving three or more variables. * *p* < 0.05; ** *p* < 0.01; **** *p* < 0.0001, ns; non-significant.

To further explore the regulatory mechanism of CEBPB on *DEFB1* transcription, we selected two highest-scoring binding regions from the previously predicted binding positions of CEBPB on the *DEFB1* promoter (Figure 3B), named site 1 and site 2 (Figure 7A). Site 1 contained two closely spaced high-scoring binding sites, named motif 1 and motif 2, while site 2 contained a single binding site named motif 3. Chromatin immunoprecipitation (ChIP) experiments confirmed the binding of CEBPB to the two regions on the *DEFB1* promoter in AEC-II cells. Overexpression of CEBPB significantly increased the binding of CEBPB to both regions (Figure 7B, *p* < 0.01; Figure S2G, *p* < 0.0001). Consistent with upregulation of CEBPB and hBD1 following H37Rv-infection, the binding of CEBPB to the *DEFB1* promoter increased in H37Rv-infected AEC-II cells compared with in cells without infection. However, only the increase in the binding to site 2 but not site 1 was observed in A549 cells (Figure 7C, *p* < 0.0001; Figure S2H, *p* < 0.0001). To further clarify the CEBPB binding sites on the *DEFB1* promoter, we generated truncations of the three motifs in site 1 and site 2, respectively or combinedly (Figure 7A), and performed luciferase reporter assays to observe the transcriptional regulation of the *DEFB1* promoter by CEBPB. The results showed that truncation of motif 1/2 not only did not decrease *DEFB1* expression but also possibly enhanced its transcriptional level. On the other hand, overexpression of CEBPB lacking motif 3 abolished the promotion of *DEFB1* expression (Figure 7D, *p* < 0.0001). Based on the results of the Chromatin immunoprecipitation (ChIP) experiments, we propose that in H37Rv-infected AEC-II cells, CEBPB primarily binds to site 2 (motif 3) on the *DEFB1* promoter to promote its transcription.

### 2.6. The ERK1/2 Pathway Regulates CEBPB Phosphorylation and in Turn DEFB1 Expression

**Figure 8:**
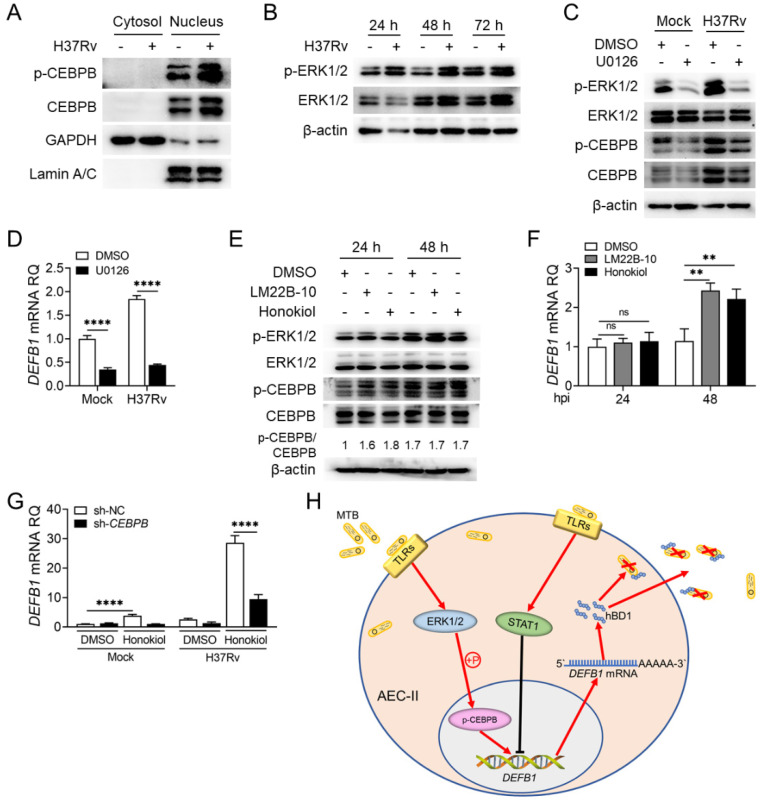
Activation of theERK1/2 pathway after Mtb infection promotes CEBPB phosphorylation and *DEFB1* expression in AEC-II cells. (**A**) Nuclear–cytoplasmic fractionation assay of the distribution of CEBPB in the nucleus and cytoplasm after infecting A549 cells with H37Rv at MOI = 10 for 72 h. (**B**) Western blot assay of activation of the ERK1/2 pathway in A549 cells infected with H37Rv at MOI = 10 for 72 h. (**C**,**D**) Western blot analysis of ERK1/2 and CEBPB phosphorylation (**C**), and qPCR analysis of *DEFB1* expression (**D**), in A549 cells pretreated with U0126 for 1 h and then infected with H37Rv at MOI = 10 for 72 h. (**E**,**F**) Western blot analysis of ERK1/2 and CEBPB phosphorylation (**E**), and qPCR analysis of *DEFB1* expression (**F**), in A549 cells pretreated with LM22B-10 or Honokiol for 1 h and infected with H37Rv at MOI = 10 for 48 h. (**G**) qPCR analysis of *DEFB1* expression in *CEBPB*-silenced A549 cells treated with Honokiol and infected with H37Rv at MOI = 10 for 48 h. (**H**) Schematic diagram of the molecular mechanism of the ERK1/2-CEBPB axis but not STAT1 in the regulation of hBD1 expression in AEC-II cells against Mtb infection. Data are presented as mean ± SD and are representative of at least three experiments with similar observations. ANOVA was used for comparison involving three or more variables. ** *p* < 0.01; **** *p* < 0.0001, ns; non-significant.

The above results demonstrated that upregulated CEBPB directly promotes *DEFB1* expression after Mtb infection. However, how H37Rv infection regulates the activity of CEBPB in AEC-II cells remains unknown. CEBPB activity is suppressed by its truncated isoform LIP, which functions as a trans-regulator by competitively binding to the same DNA recognition sequence of CEBPB. The formation of LIP is regulated by the AMPK and mTOR pathways [39,40]. Therefore, we examined the activation of the AMPK and mTOR pathways in AEC-II cells after H37Rv infection and found no significant changes in their activation (Figure S2I). Additionally, the concentrations of various CEBPB truncated isoforms were low and exhibited no significant difference before and after Mtb infection (Figure S2J), suggesting that regulation on *DEFB1* expression by CEBPB does not involve its isoforms as well as these two signaling pathways. Considering that transcription factors are usually phosphorylated in the cytoplasm before entering the nucleus to exert their activity, we firstly examined the nuclear translocation of CEBPB in AEC-II cells after H37Rv infection and found that the nuclear localization and phosphorylation of CEBPB increased following infection (Figure 8A). Since TLR stimulation after Mtb infection can activate the NF-κB and MAPK pathways and participate the anti-tuberculosis immune response [41], while Mtb can inhibit these two signaling pathways and promote Mtb survival through bacterial components such as PPE36 to suppress host innate immunity [42], we analyzed the effects of these signaling pathways on CEBPB phosphorylation. Western blotting results showed that activation of the NF-κB and MAPK pathways indeed increased following H37Rv infection (Figure 8B and Figure S3A,B). Then, we treated Mtb-infected AEC-II cells with respective inhibitors of these pathways. After confirming the inhibitory effects of the inhibitors (Figure S3C–E), we found that the NF-κB, p38 MAPK and JNK pathways exerted no significant effects on CEBPB phosphorylation and *DEFB1* expression in H37Rv-infected AEC-II cells (Figure S3F–H). Only treatment with the ERK1/2 pathway inhibitor, U0126, resulted in a significant decrease in CEBPB protein expression and phosphorylation in AEC-II cells (Figure 8C and Figure S3I), accompanied with downregulated *DEFB1* expression (Figure 8D, *p* < 0.0001; Figure S3J, *p* < 0.001). Subsequently, treatment of AEC-II cells with the ERK1/2 activators, LM22B-10 and Honokiol, respectively, resulted in increased CEBPB protein expression and phosphorylation levels, as well as increased *DEFB1* transcription. Although the significant increase was observed only at 24 h but not 48 h after treatment, the effects of these agonists are enough to promote *DEFB1* transcription due to the possible lag in transcription regulation (Figure 8E,F, *p* < 0.01). Importantly, after silencing CEBPB, Honokiol failed to effectively upregulate *DEFB1* levels (Figure 8G, *p* < 0.0001). These data indicated that in Mtb-infected AEC-II cells, the ERK1/2 pathway promotes *DEFB1* expression by enhancing CEBPB phosphorylation, thereby exerting an anti-tuberculosis function.

In summary, we found that upregulation of hBD1 in AEC-II cells is mediated by CEBPB activated by ERK1/2 following Mtb infection, but not by STAT1, and promotes anti-tuberculosis effect (Figure 8H).

## 3. Discussion

Highly expressed hBD1, the member of the defensin family, has exhibited remarkable therapeutic effects in killing bacteria, viruses, and fungi, including Mtb with remarkable efficacy [43,44,45]. It is especially noteworthy that, AMPs exert bactericidal effects through their physical properties, which make them less prone to induce drug resistance. In our study, we found that hBD1 expression was significantly upregulated in AEC-II cells after Mtb infection compared to other AMPs, such as LL-37, NHP1, etc. Significant upregulation of *DEFB1* and lacking detailed study about its function in Mtb infection prompted us to explore its role and regulation in Mtb-infected epithelial cells. For this reason, other AMPs were not assayed in this study. Functional studies showed that AEC-II cells secreting higher levels of hBD1 effectively inhibited the growth of Mtb both intracellularly and extracellularly. Conversely, cells lacking hBD1 expression or mice with the *Defb1* knockout exhibited significantly weakened anti-tuberculosis effects, highlighting the potential of hBD1 as an adjunctive anti-tuberculosis agent. A study has indicated that the defensin family members mBD3 and mBD4 play crucial roles in the early stages of tuberculosis infection and low-dose Mtb challenge. Considering the constitutive expression of hBD1 in epithelial cells, hBD1 may confer advantages in the anti-tuberculosis immune response during active pulmonary tuberculosis [46]. However, due to the high cost for its production and storage, promoting the endogenous synthesis of AMPs may be a better approach for anti-tuberculosis immunotherapy. Despite the low propensity of AMPs to induce resistance during antitubercular treatment, concerns regarding the emergence of AMPs-resistant Mtb strains have surfaced in recent years [47]. Consequently, the co-administration of AMPs alongside first-line drugs has become a common practice. Nonetheless, the therapeutic application of AMPs in the context of pulmonary tuberculosis remains challenging. Moving forward, the integration of cutting-edge synthetic biology techniques and nanoparticle delivery systems holds significant promise in unlocking the full potential of AMPs in combating tuberculosis [47]. However, motivating endogenous production of AMPs will save a lot of effort. Nevertheless, the regulatory mechanisms of hBD1 expression in Mtb-infected AEC-II cells have not been reported.

In this study, we investigated the regulatory approach of hBD1 expression in AEC-II cells. Transcriptional regulation mediated by transcription factors is the fundamental element that determines changes in molecular expression. Therefore, we used publicly available databases including JASPR and CISBP, etc., to predict the transcription factors of hBD1. Combining these predictions with our transcriptome data of AEC-II cells before and after H37Rv infection, we found five transcription factors whose expression upregulated after H37Rv infection in consistent with that of hBD1. Among them, STAT1, which plays a crucial regulatory role in Mtb infection immunity, became the focus of our study. STAT1 is an important member of the signal transducer and activator of transcription (STAT) family, and clinical case analyses have shown that human STAT1 deficiency increases susceptibility to mycobacterial infections [48,49]. Macrophages primarily use the IFN-γ/STAT1 pathway to activate the NADPH oxidase system and induces the production of inducible nitric oxide synthase, in turn producing reactive oxygen species and reactive nitrogen intermediates to kill viruses and intracellular pathogens [50]. Phosphorylated STAT1 and STAT2 mediate the IFN-α/β-receptor signaling to upregulate the transcription of more than 300 genes induced by IFN-β; nevertheless, Mtb infection can inhibit this process [51]. In this study, we observed a significant increase in STAT1 expression and phosphorylation in H37Rv-infected A549 cells, suggesting its potential role in the upregulation of hBD1 expression. However, unexpectedly, genetic- and pharmaceutical treatments demonstrated that STAT1 exerted a negative regulatory effect on *DEFB1* transcription, which is consistent with the negative correlation between *DEFB1* and *STAT1* revealed in the GSE114911 [31] dataset. HBD3, a member of the same defensin family as hBD1, induces different specific signaling cascades in various cells involved in host defense, such as MAPK activation in myeloid and keratinocytes, and STAT1 tyrosine phosphorylation and PTPase activity in T cells, which exhibiting a broad immune regulatory activity beyond conserved antibacterial activity of hBD3 and contributing to integrate innate and adaptive immunity [52]. Therefore, it is possible that upregulation of STAT1 expression and serine727 activation in H37Rv-infected A549 cells was induced by elevated hBD1, which is consistent with its immunoregulatory function. However, it seems to form a certain negative feedback regulation on the expression of hBD1, and the significance of such regulation is worth further exploration. Therefore, the transcription factor responsible for upregulating hBD1 expression in AEC-II cells after Mtb infection remains to be identified.

Subsequently, we conducted similar correlation analysis on the other four identified transcription factors and found that only CEBPB showed a positive correlation with *DEFB1* expression. CEBPB is a widely expressed transcription factor in various cells and belongs to the CCAAT/enhancer-binding protein (C/EBP) family. It is involved in multiple biological processes, including cell division, immune response, inflammation, energy metabolism, embryonic development, and adipocyte differentiation [53]. CEBPB also plays an important role in Mtb infection immunity [54]. Toshihiro Nakajima et al. found that Ras-dependent MAPK signaling specifically phosphorylates CEBPB at Thr235, inducing the expression of a variety of immune and inflammation-related genes [55]. Xu et al. identified the hsa-miR-24-3p-NEAT1-ADM-CEBPB regulatory pathway as a key network in modulating tuberculosis pathogenesis through single-cell sequencing analysis of PBMCs [54]. In macrophages, the AMPK-PPARGC1A pathway involving CEBPB upregulates multiple autophagy-related genes, promoting autophagy activation and exerting anti-tuberculosis effects [56]. These studies indicate that CEBPB is involved in immune regulation against tuberculosis infection through various pathways. However, the approach of CEBPB taking part in anti-tuberculosis immunity in AEC-II cells has not been reported.

Considering the consistency between CEBPB and *DEFB1* expression, we hypothesized that promoting AMP expression might be one of the ways in which CEBPB exerts its anti-tuberculosis immune effects. Indeed, our results indicated that CEBPB effectively upregulates hBD1 expression in AEC-II cells and inhibited the survival of Mtb. Meanwhile, both phosphorylation and nuclear translocation of CEBPB increased after Mtb infection. Interestingly, ChIP and luciferase reporter assays revealed that CEBPB could bind to two regions on the *DEFB1* promoter, referred to as site 1 and site 2, exerting different effects on *DEFB1* transcription. However, in A549 cells, we found that binding of CEBPB to motif 1 and motif 2 in site 1 may have an inhibitory effect on *DEFB1* transcription, as truncation of motif 1, motif 2, or motif 1+2 (site 1 as a whole) increased *DEFB1* transcription after H37Rv infection. Conversely, the promotion of *DEFB1* expression by CEBPB was abolished when motif 3 (site 2) was truncated. This process may involve the assistance of certain cofactors to coordinate the affinity of CEBPB binding to the two sites. Our research group is further investigating this hypothesis and examine the phenomenon in other cells. Therefore, our study demonstrates that for the same anti-tuberculosis effector, such as hBD1, there exist complex and possibly opposing regulatory mechanisms in Mtb-infected cells. For instance, STAT1, the transcription factor, typically plays a protective role in anti-tuberculosis immunity, exerted an inhibitory effect on hBD1 expression in AEC-II cells, while CEBPB, which promotes *DEFB1* transcription, exerts opposing effects depending on its binding to different sites on the *DEFB1* promoter in some cells infected with Mtb. Our study once again reveals a glimpse of the complex mechanisms underlying tuberculosis infection immunity, emphasizing the demand for comprehensive and systematic research to elucidate the mechanisms of Mtb infection immunity.

CEBPB mRNA undergoes selective splicing to generate four isoforms of proteins: full-length 38 kDa CEBPB (LAP*), 35 kDa LAP (Liver-enriched transcriptional activator protein), 21 kDa LIP (Liver-enriched transcriptional inhibitory protein), and a 14 kDa protein [6,9]. Among them, LAP and LIP are the major splice variants, while LAP* is rare [10]. LAP contains an activation domain and a basic leucine zipper domain, conferring its full transcriptional regulatory activity. On the other hand, LIP only consists of the basic leucine zipper domain and acts as a transcriptional repressor by forming inactive heterodimers with other family members. The relative expression levels of LAP and LIP can indicate whether CEBPB functions as a transcriptional activator or inhibitor. The production of LAP and LIP is regulated by the AMPK and mTOR pathways. Glycolysis inhibits AMPK-ULK1 signaling and autophagy formation, leading to reduced autophagy-mediated LAP reduction. LAP, in turn, enhances G-CSF expression and supports the development of myeloid-derived suppressor cells in tumors. In the mTOR pathway, the phosphorylation of 4E-BP1 and subsequent inhibition of eIF4E can suppress LIP formation mediated by autophagy, indirectly promoting LAP activity. To investigate whether the transcriptional activity of CEBPB on *DEFB1* is regulated by the AMPK and mTOR pathways and the resulting truncation of CEBPB, we first examined the activation of AMPK and mTOR in AEC-II cells after H37Rv infection. Our results showed no significant differences in the activation of these pathways before and after infection during our observation period. Consistent with this, the levels of CEBPB LIP were very low in cells no matter with or without infection, showing no significant differences among different treatments. This is inconsistent with the observed increase in CEBPB expression and its binding to the *DEFB1* promoter after H37Rv infection. Therefore, this pathway is not the main mechanism regulating the transcriptional activity of CEBPB on *DEFB1* in AEC-II cells after H37Rv infection.

So, how is the activity of CEBPB in regulating *DEFB1* transcription controlled in Mtb-infected AEC-II cells? The activity of transcription factors is often regulated by their own expression levels and post-translational modifications, especially phosphorylation. Activation of the ERK1/2 pathway significantly inhibits the degradation of CEBPB mediated by the ubiquitin ligase COP1 [57]. Rebecca Chinery et al. found that PKA phosphorylates CEBPB at Ser299, promoting the upregulation of the cell cycle regulatory protein p21 and inducing apoptosis in cancer cells [58]. Through a literature review, we found that the transcriptional activity of CEBPB can be regulated by various signaling pathways, including MAPK and NF-κB, which are important in the regulation of anti-tuberculosis immune response. Therefore, we attempted to use inhibitors of these pathways to treat A549 cells, and found that only inhibition of the ERK1/2 pathway led to a decrease in CEBPB phosphorylation levels and a significant downregulation of *DEFB1* transcription. This suggests that in Mtb-infected AEC-II cells, the ERK1/2 pathway mediates the phosphorylation of CEBPB, thereby upregulating hBD1 expression to exert its anti-tuberculosis effect. Although in our study only the regulation of hBD1 expression was explored, the above activation pathway of CEBPB may possibly function for other members of the defensin family, because CEBPB has been previously reported to regulate hBD2 in oral epithelial cells or oral keratinocyte cells [59]. It is interesting to explore the tissue or cell specificity of CEBPB on expression of different membranes of the defensin family.

In summary, our study demonstrated that increased hBD1 expression during Mtb infection can inhibit the survival of Mtb, and CEBPB can be phosphorylated by ERK1/2, effectively promoting hBD1 expression and exerting anti-tuberculosis effects. Our research supplements the function of hBD1 in anti-tuberculosis immunity and elucidates the regulatory mechanism of hBD1 expression in Mtb-infected AEC-II cells, providing a basis for the development of novel immunotherapies against tuberculosis and offering new insights for the treatment of drug-resistant tuberculosis. It is worth noting that the transcriptional regulation mechanism of hBD1 discovered in this study has only been validated in vitro cell models, and further validation in experimental animals is needed before exploring clinical applications.

## 4. Materials and Methods

### 4.1. Cells, Mice, and Agents

The use of PBMCs from tuberculosis patients was approved by the Ethics Committee of Guangzhou Chest Hospital. PBMCs from healthy volunteers were obtained from the Guangzhou Blood Center, the sociodemographic characteristics of the patients are provided in Supplementary Table S1.

*C57BL/6J* mice under SPF conditions were obtained from the Experimental Animal Management Center of Southern Medical University. The experimental protocol was approved by the Biosafety Management Committee and Medical Ethics Committee of Southern Medical University. *Defb1*^−/−^ mice were purchased from Guangzhou Saiye Company (Guangzhou, China). Transgenic mice were genotyped using PCR and standard agarose gel electrophoresis following tail tissue DNA extraction as per the manufacturer’s instruction (Omega Bio Tek Inc., Norcross, GA, USA). The primer sequences are provided in Supplementary Table S2. All mice were housed and maintained at the Experimental Animal Management Center of Southern Medical University.

A549 cells (Cat#: CL-0016, Pricella, Wuhan, China) and BEAS-2B cells (Cat#: CRL-9609, ATCC) were cultured in DMEM (Corning Inc., Manassas, VA, USA) supplemented with 10% fetal bovine serum (Corning Inc.).

As described in the figure legends, cells were treated with the following reagents: IFN-γ (20 ng/mL; T&L Biotechnology, Beijing, China), Fludarabine (10 μM), U0126 (20 μM), JSH-23 (10 μM), SP600125 (20 μM), SB203580 (10 μM), LM22B-10 (1 μM) (Selleck cn. Inc., Shanghai, China), Honokiol (100 μM; MedChemExpress LLC. (MCE), Monmouth Junction, NJ, USA).

### 4.2. Mtb Culture, Infection, and Colony-Forming Unit Assay (CFU)

The standard strain H37Rv of Mtb was cultured in Difco^TM^ Middlebrook 7H9 medium (BD Biosciences, San Jose, CA, USA) supplemented with a 1/9 volume ratio of oleic acid albumin dextrose catalase (OADC). The culture was maintained in a 37 °C incubator with 5% CO_2_. For the experiment, a logarithmic growth phase suspension of the strain was obtained by centrifugation at 1500× *g*. The pellet was resuspended in complete DMEM culture medium and thoroughly homogenized by repeated grinding (30–50 times). The resulting homogenate containing single bacteria was then centrifuged at 1500× *g* for 5 min. The supernatant was collected, and the OD 600 nm was measured using a biophotometer plus spectrophotometer (Eppendorf, Hamburg, Germany). A bacterial suspension with an OD value of 0.207 corresponded to a concentration of 4 × 10^6^ colonies/mL. For subsequent experiments, RNA and proteins were collected at a time corresponding to infecting AEC-II cells at a multiplicity of infection (MOI) of 5. The number of bacterial colonies inside and outside the cells was determined using CFU assays.

### 4.3. RNA Extraction, Quantitative Real-Time PCR, and High-Throughput RNA Sequencing

Total RNA was extracted using TranZol reagent (TransGen Biotech Inc., Beijing, China) and the concentration and purity of RNA were determined using a NanoDrop 2000 UV-Vis spectrophotometer (Thermo Fisher). The extracted RNA underwent gDNA removal and cDNA reverse transcription using the TransScript One Step gDNA Removal and cDNA Synthesis SuperMix kit (Transgen Biotech, Beijing, China). The resulting cDNA was subjected to quantitative real-time PCR (qPCR) using the TransStart Top Green qPCR SuperMix kit (Transgen Biotech) on a LightCycler96 instrument (Roche Ltd., Basel, Switzerland). The expression of β-actin was used as a reference for normalization, and the 2^−ΔΔCT^ method was employed to quantify the abundance of target mRNA. For high-throughput RNA sequencing, cell lysates were prepared using TRansZol reagent and sequenced on an Illumina HiSeq 2500 platform at Guangzhou Ruibo Biotechnology Co., Ltd. (Guangzhou, China). The RNA-seq data were aligned to the human reference genome sequence (UCSC hg38 assembly) using hisat2. The primer sequences are listed in Supplementary Table S2.

### 4.4. Recombinant Plasmid Construction and Nucleic Acid Transfection

Nucleic acid segments encoding hBD1 and CEBPB were cloned into the recombinant pLVX/CMV-3×Flag lentivirus plasmid and packaged to recombinant lentiviruses in 293T cells (Cat#: CRL-3216, ATCC). The obtained LV-*DEFB1* and LV-*CEBPB* were used to infect A549 and BEAS-2B cells followed by purinomycin (Thermo Fisher Scientific Inc., Rockford, IL, USA) selection. Nucleic acid segments encoding STAT1 and CEBPB were cloned into the eukaryotic expression plasmid pcDNA3.1 with 3×Flag tag at C terminal. Recombinant pcDNA3.1 plasmids were transfected into 293T, A549 and BEAS-2B cells using polyethylenimine linear (PEI) MW40000 (Yeasen Biotechnology (Shanghai) Co., Ltd., Shanghai, China), following the manufacturer’s instructions.

Small RNAs targeting human *STAT1* and a scramble oligonucleotide (si-NC) were synthesized (Guangzhou Ribio Co., Ltd., Guangzhou, China) and then transfected into A549 cells using Lipofectamine™ 2000 (Thermo Fisher) according to the protocol of the manufacturer. shRNAs targeting human *CEBPB* and a non-targeting control oligonucleotide were also synthesized and cloned into the recombinant pLVX/U6 lentivirus plasmid. The recombinant LV-sh*CEBPB* were used to infect A549 cells, followed by purinomycin selection. Forty-eight hours later, the overexpression and silence efficiency were detected using qPCR and Western blotting before further experiments.

To knockout the *DEFB1* gene in A549 cells, the CRISPR-Cas9 method was employed according to the procedure described previously [60]. Following sequencing and knockout efficiency identification, the stable *DEFB1*^−/−^ A549 cell strain was obtained through purithromycin screening.

Full-length, ∆1-∆5 truncation forms of the *DEFB1* promoter were cloned into the pGL4.10 plasmids and were transfected into 293T cells using PEI.

The related nucleic acid sequences are all provided in Supplementary Table S2.

### 4.5. Animal Experiments

To investigate the role of hBD1 in anti-Mtb infection, wild-type (WT, n = 5) and *Defb1^−/−^* mice (n = 5) were exposed to an aerosolized suspension of H37Rv at a concentration of 10^6^ colony-forming units (CFU) using an aerosol generator (Glass Cool, LLC, Terre Haute, IN, USA) for 24 h. Mice were euthanized at 1, 4 and 8 weeks post-infection. Approximately 200 μL of peripheral blood was collected, centrifuged at 3000 rpm for 5 min, and stored at −80 °C for subsequent analysis using the ProcartaPlex Mix&Match Luminex assay (Thermo Fisher). Lung tissues were partially lysed with 0.2% Triton–PBS to determine bacterial load using the CFU assay. The remaining lung tissue was fixed with 4% paraformaldehyde in PBS, embedded in paraffin, and sectioned into 5 μm thick slices. Hematoxylin and eosin (H&E) staining was performed to evaluate tissue inflammation and injury. In the process of animal experiments, we strictly followed the ARRIVE guidelines 2.0 [61], and adhered to the 3R principles and 5 freedoms to ensure animal welfare and alleviate animal suffering. Regarding animal welfare, we adhere to the 3Rs principle—replacement, reduction, and refinement—ensuring the five freedoms for mice—freedom from hunger and thirst; discomfort; pain, injury, and disease; to express normal behavior; and from fear and distress.

### 4.6. Western Blotting and Subcellular Fractionation

Cells were harvested by washing with cold 1 × PBS and lysed using RIPA buffer (20 mM HEPES, pH 7.9, 400 mM NaCl, 1 mM EDTA, 10 mM KCl, 20% glycerol, 1% NP-40, 0.5% sodium deoxycholate, and 0.1% sodium dodecyl sulfate) supplemented with 1/10 volume of PhosSTOP phosphatase inhibitor (Roche) and 1/10 volume of protease inhibitor mixture (Roche), and 1 mM DTT (Biosharp, Hefei, China). Protein quantification was performed using the Bradford reagent (Bio Rad Laboratories Co., Ltd., Hercules, CA, USA). The lysates were subjected to SDS-PAGE electrophoresis and transferred onto a PVDF membrane (Merck Millipore Ltd., Tullagreen, Carrigtwohill, Co., Cork, Ireland). After blocking with skim milk powder, the following primary antibodies were incubated. Unless specified, the antibody dilution was 1:1000: β-actin (Cat# 8457), GAPDH (Cat# 2118S), phospho-CEBPB (Cat# 3084), p44/42 MAPK (Erk1/2) (Cat# 4695S), phospho-p44/42 MAPK (Erk1/2) (Cat# 4370S), SAPK/JNK (Cat# 9252S), phospho-SAPK/JNK (Cat# 9251S), p38 MAPK (Cat# 8690S), phospho-p38 MAPK (Cat# 4511S), Lamin A/C (Cat#: 4777) (Cell Signaling Technology, Inc., Beverly, MA, USA), FLAG^®^ M2 (Cat#: F1804, Sigma-Aldrich, Darmstadt, Germany), CEBPB (Cat#: 23431-1-AP, Proteintech Group, Inc, Rosemont, IL, USA). Corresponding horseradish peroxidase (HRP)-conjugated secondary antibodies (1:5000; goat anti-mouse IgG (H+L), Cat#: 31430; goat anti-rabbit IgG F(ab’)2, Cat#: 31234; ThermoFisher) were used. Signal detection was performed using FDbio Pico ECL (Hangzhou Fude Biotechnology Co., Ltd., Hangzhou, China) and visualized using FluorChem (ProteinSimple, Wallingford, CT, USA).

To investigate the cellular localization of CEBPB, A549 cells were subjected to nucleocytoplasmic separation using a nuclear protein and cytoplasmic protein extraction kit (Beyotime Inc., Shanghai, China) following the manufacturer’s instructions. The levels of CEBPB in the obtained fractions were analyzed by Western blotting, with GAPDH and Lamin A/C serving as reference proteins for the cytoplasm and nucleus, respectively.

### 4.7. Luciferase Reporter Assay

pcDNA3.1-*STAT1*, pcDNA3.1-*CEBPB*, pGL4.10-*DEFB1*-promote and pRL-TK were transfected into 293T using PEI, with corresponding empty vectors as controls. According to the manufacturer’s protocol, at 48 h after transfection, Dual Glo^®^ Luciferase Assay System (Promega (Beijing) Biotech Co., Ltd., Beijing, China) was used to measure luciferase activity, and the signal was obtained with the Spark Cyto imaging cytometer (Tecan Trading AG, Männedorf, Switzerland). The difference in transfection efficiency was corrected by normalizing firefly luciferase activity to the total Renilla luciferase activity.

### 4.8. Chromatin Immunoprecipitation (ChIP)

To validate the binding sites of the *DEFB1* promoter by CEBPB, ChIP experiments were performed following a previously reported protocol [62] with the following modifications. After the corresponding treatment, 10^7^ of A549 or BEAS-2B cells were collected and resuspended in ChIP lysis buffer (50 mM Tris-HCl PH8.1, 10 mM EDTA, 1% SDS, supplemented with 1/10 protease inhibitor) at 4 °C for 20 min. Subsequently, the sample was sonicated (15 cycles, 30 s on/30 s off, 4 °C) and centrifuged at 4 °C and 12000 rpm for 10 min. The supernatant was collected and quantified. Next, 10 mg of protein and 5 μg of antibodies were co-incubated at 4 °C overnight and mixed with protein A/G magnetic beads (MCE) at 4 °C for 2 h. The magnetic bead–protein–antibody complex was washed 5 times with the Low salt wash buffer (20 mM Tris-HCl pH8.1, 150 mM NaCl, 2 mM EDTA, 0.1% SDS, and 1% Triton X-100), high salt wash buffer (20 mM Tris-HCl pH8.1, 500 mM NaCl, 2 mM EDTA, 0.1% SDS, and 1% Triton X-100), LiCl wash buffer (10 mM Tris-HCl pH8.1, 0.25 M LiCl, 1 mM EDTA, 1% deoxycholate, and 1% NP-40), and TE buffer (10 mM Tris-HCl pH8.1, and 1 mM EDTA) on a magnetic rack. The protein complex was resuspended on the Elution buffer (1% SDS, 0.1 M NaHCO_2_) and separated from the magnetic beads, and 10 μL of 5 M NaCl was added for de-crosslink at 65 °C for 4 h. Subsequently, 1 μL of 20 mg/mL RNase A (Omega Biotek Inc., Norcross, GA, USA) was added and incubated at 37 °C for 30 min. The samples were treated with 1 μL of sterile water containing 2 μg/mL protease K (Merck Millipore) without RNase and incubated at 55 °C for 50 min. The co-precipitated DNA was then separated according to the FastPure Gel DNA Extraction Mini Kit (Vazyme, Nanjing, China) manufacturer’s instructions, dissolved in 20 μL of water, and subjected to qPCR analysis. The relative enrichment was calculated as the ratio of the amplified DNA value obtained after p-CEBPB immunoprecipitation normalized to that after normal IgG immunoprecipitation, then relative to the value of the vector group without infection, which was set to 1. The primers used in the qRT-PCR are listed in Supplementary Table S2.

### 4.9. Statistical Analysis

Representative data from a minimum of three independent experiments are presented as mean ± standard deviation (SD). When comparing two parameters, a *t*-test was used to assess the differences. For comparisons involving more than three parameters, one-way ANOVA was employed to analyze the differences in response variables affected by the factors. Two-way ANOVA was utilized to assess the impact of two independent factors on the response variable and determine whether there was an interaction between the two factors. Post hoc multiple comparisons were performed using the Least Significant Difference (LSD) or Dunnett’s T3 method. A significance level of *p* < 0.05 was considered statistically significant. All statistical analyses were conducted using GraphPad Prism 9.4.1 (San Diego, CA, USA).

## Data Availability

RNA-seq data have been submitted in NCBI Sequence Read Archive database (https://www.ncbi.nlm.nih.gov/sra, accessed on 17 October 2023) with the accession code PRJNA1028980. All data generated or analyzed during this study are included in this published article. The data that support the findings of this study are available on request from the corresponding authors.

## Supplementary Materials

The following supporting information can be downloaded at: https://www.mdpi.com/article/10.3390/ijms25042408/s1.
