# Supplementary material for: ERK1/2-CEBPB Axis-Regulated hBD1 Enhances Anti-Tuberculosis Capacity in Alveolar Type II Epithelial Cells
Source: Int J Mol Sci. 2024 Feb 18;25(4):2408. doi: 10.3390/ijms25042408 (PMC10889425; doi:10.3390/ijms25042408)
Supplement: Supplementary file 1 [file ijms-25-02408-s001.zip › 3-Supplementary Table.pdf]

Supplementary Table 1. Patient information

Group	No.	Gender	Age
Healthy controls	1	Male	22
	2	Female	73
	3	Female	24
	4	Male	15
	5	Female	22
	6	Female	15
	7	Female	38
	8	Male	30
	9	Male	21
	10	Female	16
	11	Female	23
	12	Female	27
	13	Male	23
	14	Male	20
	15	Male	43
	16	Male	22
	17	Female	59
	18	Male	29
	19	Male	38
	20	Female	32
	21	Female	14
	22	Male	32
	23	Female	59
	24	Male	40
	25	Female	38
	26	Female	62
	27	Female	29
	28	Male	16
	29	Female	28
	30	Female	27
	31	Male	35
	32	Male	45
	33	Female	28
	34	Male	60

Pulmonary tuberculosis patients	1	Male	59
	2	Male	30
	3	Female	43
	4	Male	62
	5	Female	30
	6	Female	60
	7	Female	19
	8	Male	48
	9	Female	30
	10	Male	20
	11	Female	28
	12	Female	27
	13	Male	25
	14	Female	24
	15	Male	21
	16	Female	25
	17	Female	58
	18	Female	51
	19	Male	59
	20	Male	48
	21	Female	25
	22	Female	25
	23	Male	59
	24	Male	25
	25	Male	38

Supplementary Table 2. Sequences of siRNA and primers

siRNA		
**SiRNA**	**Targeting sequence**	**Accession number**
sh-*CEBPB*-1	5’-CAUCGACUUCAGCCCGUACCUTT-3’	NM_005194.4
sh-*CEBPB*-2	5’-GCCCTGAGTAATCGCTTAA-3’	
si-*STAT1*-1	5’-GCAGCACAACATACGGAAA-3’	XM_054343560.1
si-*STAT1*-2	5’-GCTGAACTATAACTTGAAA-3’	

qPCR primers			
**Gene**	**Primer pairs**	**Amplicon**	**Accession number**
Actin	FP: 5’-TCAAGATCATTGCTCCTCCTGAG-3’<br>RP: 5’-ACATCTGCTGGAAGGTGGACA-3’	87 bp	NM_00110<br>1.5
DEFB1	FP: 5’-GGTAACTTTCTCACAGGCCTTGG-3’<br>RP: 5’-TCCCTCTGTAACAGGTGCCTTG-3’	124 bp	NM_00521<br>8.4
CEBPB	FP: 5’-AGAAGACCGTGGACAAGCACAG-3’<br>RP: 5’-CTCCAGGACCTTGTGCTGCGT-3’	125 bp	NM_00519<br>4.4
STAT1	FP: 5’-ATGGCAGTCTGGCGGCTGAATT-3’<br>RP: 5’-CCAAACCAGGCTGGCACAATTG-3’	144 bp	XM_05434<br>3560.1
CHIP site 1	FP: 5’-CCACTGCGCCCTGACC-3’<br>RP: 5’-TGGGGAGGCTTCCAGGTTAT-3’	220 bp	NC_000008<br>.11
CHIP site 2	FP: 5’-TGCTCAGGGCAAATGACACC-3’<br>RP: 5’-ACCCAGGATTTCAGGAACTGG-3’	193 bp	NC_000008<br>.11

PCR primers for human identification		
**Gene**	**Primer pairs**	**Amplicon**
Defb1-sg-F1	5’-TGGGCCTTCATAGGTAACCTGTC-3’	Wt: none<br>KO: 559 bp
Defb1-sg-R1	5’-AAACTACTACATCATGTGTGCCATC-3’	
Defb1-wt-F2	5’ -TGGGCCTTCATAGGTAACCTGTC-3’	Wt: 654 bp<br>KO: none
Defb1-wt-R2	5’-GAATGGCTCAATACATTTGACGCTC-3’	

PCR primers for plasmid construction		
**Gene**	**Primer pairs**	**Amplicon**
DEFB1	FP: 5’-CGGGATCCATGAGAACTTCCTACCTTCT-3’<br>RP: 5’-CGTCTAGACTTGCAGCACTTGGCCT-3’	220 bp
CEBPB	FP: 5’-CGGAATTCATGCAACGCCTGGTGG-3’<br>RP: 5’-CGGGATCCGCAGTGGCCGGAGGA-3’	1051 bp
STAT1	FP: 5’-CGGCTAGCGCCACCATGTCTCAGTGGTACGAACTTC-3’<br>RP: 5’-CGGGATCCCGTACTGTGTTCATCATACTGTCGAAT-3’	2274 bp
DEFB1-<br>promoter FL	FP: 5’-CGGGTACCACCACACCCAGCTAATTTTTG-3’<br>RP: 5’-CCAAGCTTAAGTAAGCAGAGAGTAAACAG-3’	2201 bp
DEFB1-<br>promoter Δ1	FP: 5’-CGGGTACCAGCTTGAAGGTGCCTGAGATATT-3’<br>RP: 5’-CCAAGCTTAAGTAAGCAGAGAGTAAACAG-3’	1928 bp
DEFB1-<br>promoter Δ2	FP: 5’-CGGGTACCAATAACCTGGAAGCCTCCCC-3’<br>RP: 5’-CCAAGCTTAAGTAAGCAGAGAGTAAACAG-3’	1869 bp

DEFB1-promoter Δ3	FP: 5’-CGGGTACCAATAACCTGGAAGCCTCCCC-3’<br>5’-CCAAGCTTAATCGCTAACCCCTGGTGTC-3’	1651 bp
DEFB1-promoter Δ4	FP: 5’-CGGGTACCACCACACCCAGCTAATTTTTG-3’<br>RP: 5’-CCAAGCTTAATCGCTAACCCCTGGTGTC-3’	1983 bp
DEFB1-promoter Δ5	FP: 5’-CGGGTACCAGCTTGAAGGTGCCTGAGATATT-3’<br>RP: 5’-CCAAGCTTAATCGCTAACCCCTGGTGTC-3’	1710 bp
**gDNA for *DEFB1* knockout with CRISPR-Cas9**		
**Gene**	**gDNA**	**Amplicon**
DEFB1-sg-top	5’-CACCGAAGTTCTCATGGCGACTGGC-3’	21 bp
DEFB1-sg-bottom	5’-AAACGCCAGTCGCCATGAGAACTTC-3’	
