# Supplementary material for: ERK1/2-CEBPB Axis-Regulated hBD1 Enhances Anti-Tuberculosis Capacity in Alveolar Type II Epithelial Cells
Source: Int J Mol Sci. 2024 Feb 18;25(4):2408. doi: 10.3390/ijms25042408 (PMC10889425; doi:10.3390/ijms25042408)
Supplement: Supplementary file 1 [file ijms-25-02408-s001.zip › 2-supplementary fig.pdf]

Supplementary Figures for

ERK1/2-CEBPB axis-regulated hBD1 enhances anti-tuberculosis capacity in alveolar type II epithelial cells

S1 Fig.

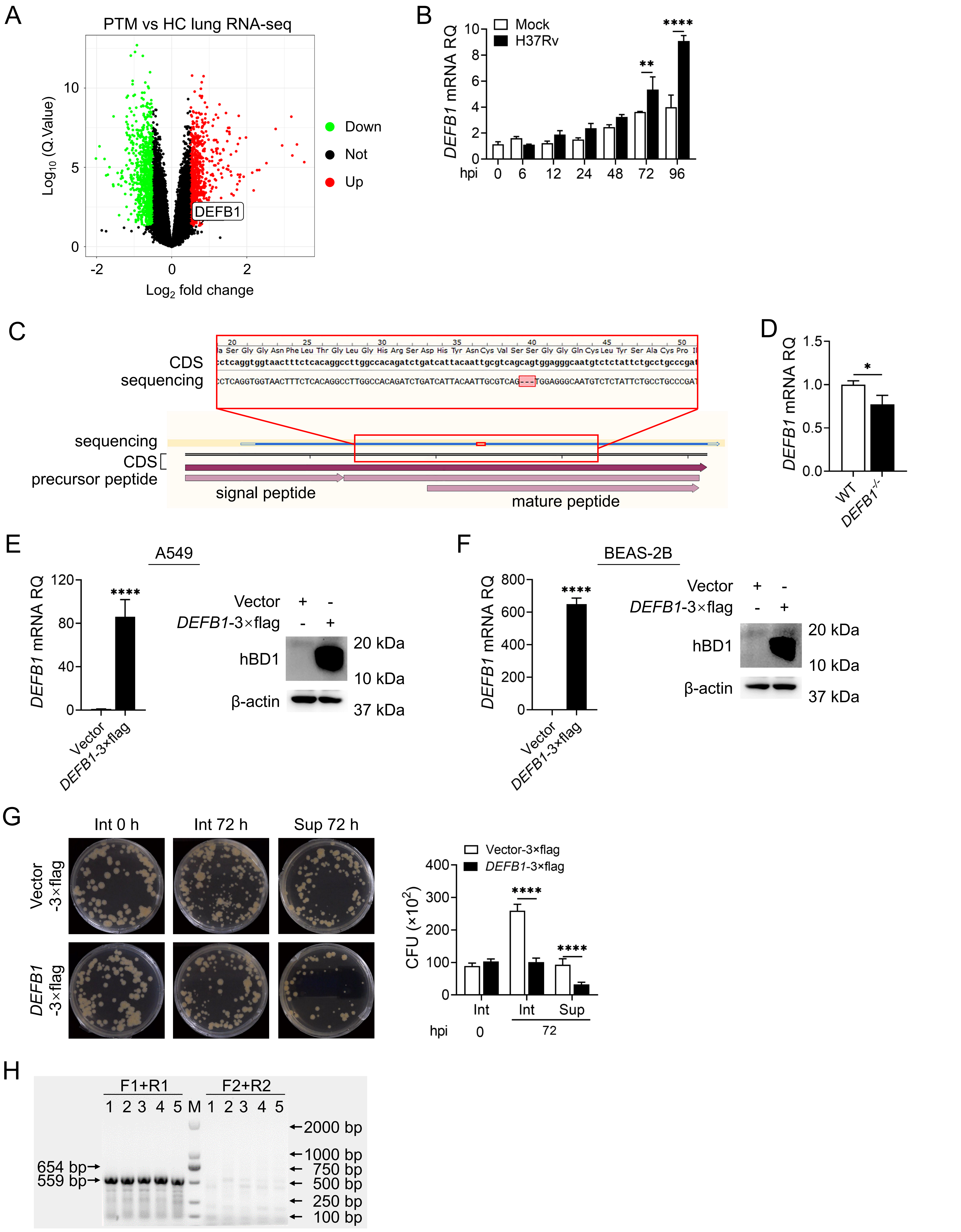
A
PTM vs HC lung RNA-seq
Down
Not
Up
DEFB1
Log10 (Q.Value)
Log2 fold change
B
Mock
H37Rv
DEFB1 mRNA RQ
hpi
C
CDS sequencing
sequencing
CDS
precursor peptide
signal peptide
mature peptide
D
DEFB1 mRNA RQ
WT
DEFB1-/-
E
A549
Vector
DEFB1-3×flag
hBD1
β-actin
20 kDa
10 kDa
37 kDa
F
BEAS-2B
G
Int 0 h
Int 72 h
Sup 72 h
Vector -3×flag
DEFB1 -3×flag
Vector-3×flag
DEFB1-3×flag
CFU (×10²)
H
F1+R1
F2+R2
654 bp
559 bp
2000 bp
1000 bp
750 bp
500 bp
250 bp
100 bp

S1 Fig. Upregulation of hBD1 reduces the *Mycobacterium tuberculosis* (Mtb) load in Type II alveolar epithelial cells (AEC-II) cells. (A) The volcano plot for visualizing differentially expressed genes in the whole lung tissues between patients infected with tuberculosis (PTM) and uninfected individuals (HC) from GEO (GSE114911 dataset) [27] identified according to the criteria of $\log_2$ fold change > 0.5 and *P*-value < 0.05. (B) qPCR analysis of *DEFB1* expression in BEAS-2B cells infected with H37Rv at multiplicity of infection (MOI) = 5 for 96 h. (C-D) qPCR and western blot analysis of hBD1 expression in A549 cells (C) and BEAS-2B cells (D) infected with LV-*DEFB1*-3×flag. (E) colony forming unit (CFU) assays of intracellular (Int) and extracellular (Sup) bacterial loads in BEAS-2B cells overexpressing *DEFB1* and infected with H37Rv at MOI = 10 after 72 h. (F) Sequencing analysis of *DEFB1* gene knockout in A549 cells using CRISPR-Cas9. (G) qPCR analysis of *DEFB1* expression in *DEFB1*$^{-/-}$ A549 cells. (H) The genotyping of C57BL/6J *Defb1*$^{-/-}$ mice was conducted using nucleic acid electrophoresis. Data are presented as mean ± SD and are representative of at least three experiments with similar observations. Simple *t*-tests and ANOVA were used for comparisons involving two and three or more variables, respectively. *$P < 0.05$; **$P < 0.01$; ***$P < 0.001$; ****$P < 0.0001$.

S2 Fig.

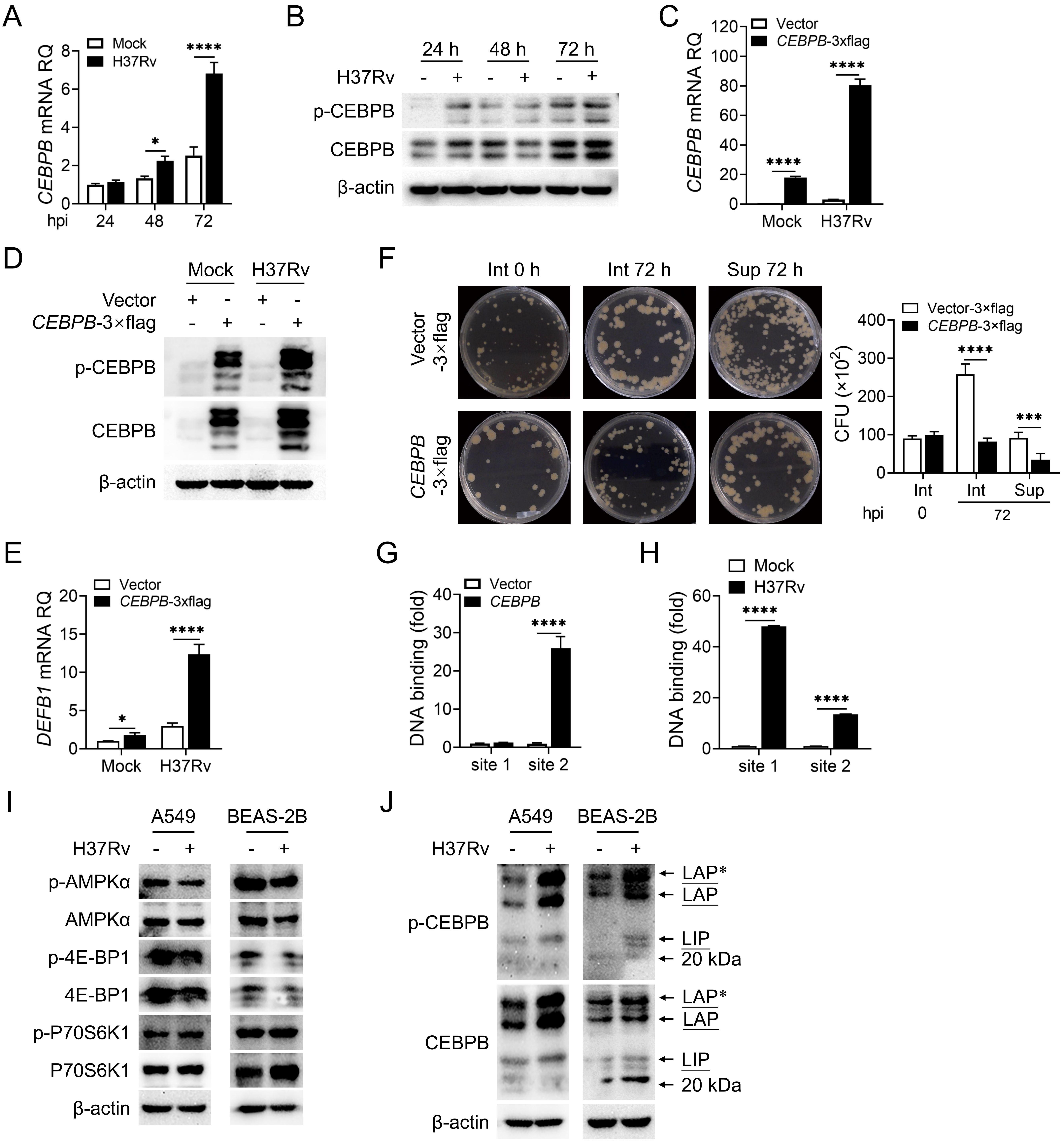
A
CEBPB mRNA RQ
Mock
H37Rv
hpi
24
48
72
B
24 h
48 h
72 h
H37Rv
p-CEBPB
CEBPB
β-actin
C
Vector
CEBPB-3xflag
CEBPB mRNA RQ
Mock
H37Rv
D
Mock
H37Rv
Vector
CEBPB-3×flag
p-CEBPB
CEBPB
β-actin
F
Int 0 h
Int 72 h
Sup 72 h
Vector -3×flag
CEBPB -3×flag
Vector-3×flag
CEBPB-3×flag
CFU (×10²)
Int
Int
Sup
hpi
0
72
E
Vector
CEBPB-3xflag
DEFB1 mRNA RQ
Mock
H37Rv
G
Vector
CEBPB
DNA binding (fold)
site 1
site 2
H
Mock
H37Rv
DNA binding (fold)
site 1
site 2
I
A549
BEAS-2B
H37Rv
p-AMPKα
AMPKα
p-4E-BP1
4E-BP1
p-P70S6K1
P70S6K1
β-actin
J
A549
BEAS-2B
H37Rv
p-CEBPB
CEBPB
β-actin
LAP*
LAP
LIP
20 kDa

S2 Fig. CEBPB directly regulates DEFB1 expression, independent of its truncated isoforms. (A-B) qPCR and western blot analysis of CEBPB mRNA (A) and protein (B) expression in BEAS-2B cells infected with H37Rv at an MOI = 5 for 72 h. (C-E) qPCR and western blot analysis of CEBPB (C-D) and *DEFB1* (E) expression in BEAS-2B cells overexpressed with CEBPB. (F) CFU assays of intracellular (Int) and extracellular (Sup) H37Rv load in BEAS-2B cells overexpressing CEBPB. (G) Chromatin immunoprecipitation (ChIP) assays of CEBPB binding to the different regions of *DEFB1* promoter in BEAS-2B cells with CEBPB overexpression. (H) ChIP assay of CEBPB binding to the different regions of *DEFB1* promoter in BEAS-2B cells infected with H37Rv at MOI = 5 for 72 h. (I) Western blot analysis of activation of AMPK and mTOR pathways in A549 and BEAS-2B cells infected with H37Rv for 72 h. (J) Western blot analysis of CEBPB phosphorylation and its truncated isoforms in A549 and BEAS-2B cells infected with H37Rv for 72 h. Data are presented as mean ± SD and are representative of at least three experiments with similar observations. ANOVA was used for comparison involving three or more variables. *$P < 0.05$; **$P < 0.01$; ***$P < 0.001$; ****$P < 0.0001$.

S3 Fig.

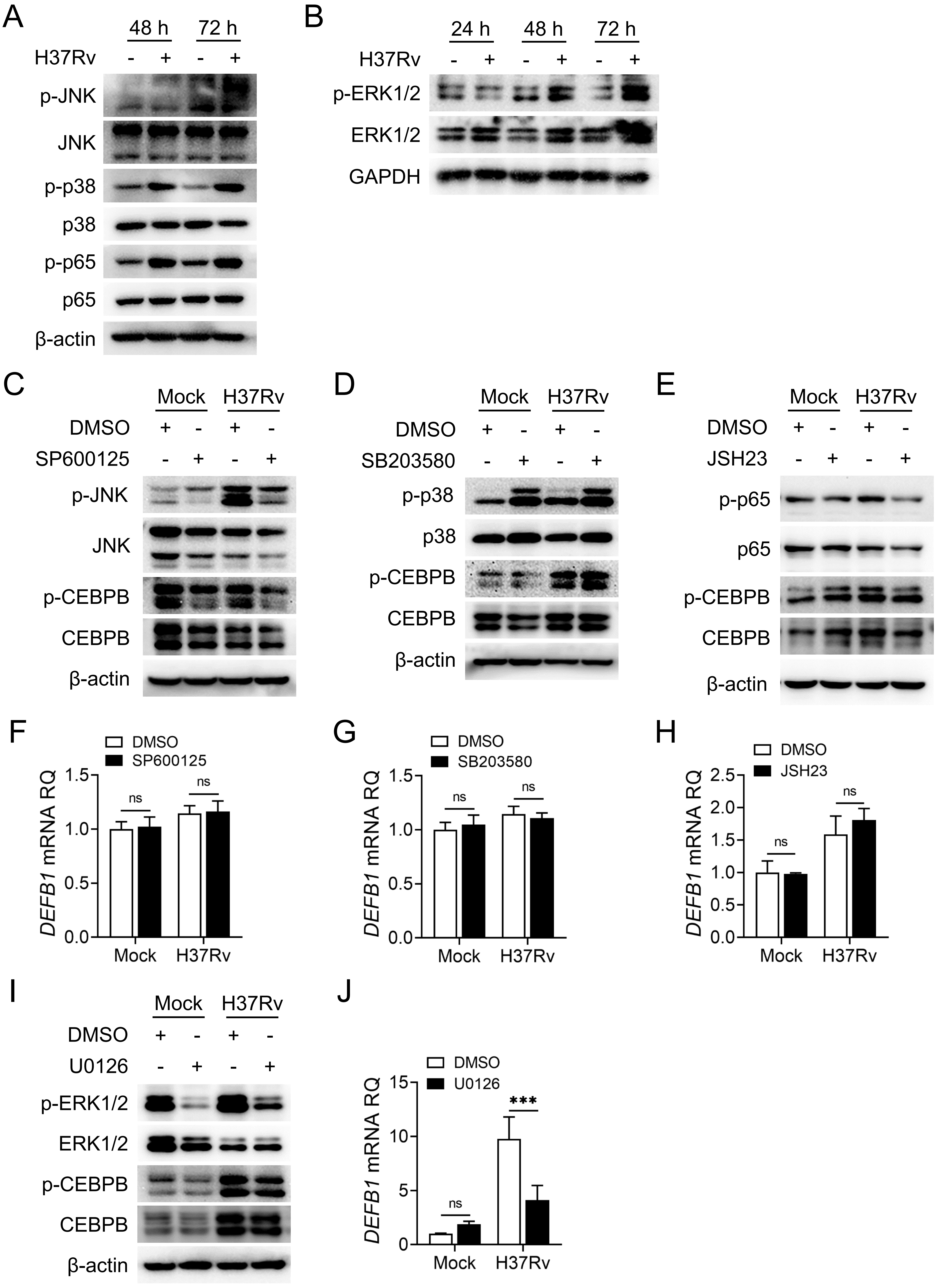
A
48 h
72 h
H37Rv - + - +
p-JNK
JNK
p-p38
p38
p-p65
p65
β-actin
B
24 h
48 h
72 h
H37Rv - + - + - +
p-ERK1/2
ERK1/2
GAPDH
C
Mock
H37Rv
DMSO + - + -
SP600125 - + - +
p-JNK
JNK
p-CEBPB
CEBPB
β-actin
D
Mock
H37Rv
DMSO + - + -
SB203580 - + - +
p-p38
p38
p-CEBPB
CEBPB
β-actin
E
Mock
H37Rv
DMSO + - + -
JSH23 - + - +
p-p65
p65
p-CEBPB
CEBPB
β-actin
F
DMSO
SP600125
DEFB1 mRNA RQ
ns
ns
Mock
H37Rv
G
DMSO
SB203580
DEFB1 mRNA RQ
ns
ns
Mock
H37Rv
H
DMSO
JSH23
DEFB1 mRNA RQ
ns
ns
Mock
H37Rv
I
Mock
H37Rv
DMSO + - + -
U0126 - + - +
p-ERK1/2
ERK1/2
p-CEBPB
CEBPB
β-actin
J
DMSO
U0126
DEFB1 mRNA RQ
ns
***
Mock
H37Rv

S3 Fig. Screening of signaling pathways regulating CEBPB phosphorylation and DEFB1 expression in AEC-II cells. (A) Western blot analysis of activation of JNK, p38 and p65 in A549 cells infected with H37Rv at MOI = 5 for 72 h. (B) Western blot analysis of activation of ERK1/2 in BEAS-2B cells infected with H37Rv at MOI = 5 for 72 h. (C-E) Western blot analysis of CEBPB phosphorylation and activation of JNK (C), p38 (D) and p65 (E) in A549 cells treated with SP600125, SB203580 and JSH23, respectively, followed by H37Rv infection at MOI = 10 for 72 h. (F-H) qPCR analysis of *DEFB1* expression in A549 cells treated with SP600125 (F), SB203580 (G) and JSH23 (H), respectively, followed by H37Rv infection at MOI = 10 for 72 h. (I-J) Western blot analysis of CEBPB phosphorylation and activation of ERK1/2 (I) and qPCR analysis of *DEFB1* expression (J) in BEAS-2B cells treated with U0126 followed by H37Rv infection at MOI = 10 for 72 h. Data are presented as mean ± SD and are representative of at least three experiments with similar observations. ANOVA was used for comparison involving three or more variables. *$P < 0.05$; **$P < 0.01$; ***$P < 0.001$; ****$P < 0.0001$.
